# The Life of John Collins Warren, M. D., Compiled Chiefly from His Autobiography and Journals

**Published:** 1860-06

**Authors:** 


### BOOK AND PAMPHLET NOTICES.

THE LIFE OF JOHN COLLINS WARREN, M. D.: Compiled chiefly from his Autobiography and Journals By EDWARD WARREN, M. D., in two volumes, Octavo. Boston: Ticknor and Fields. 1860.

This is the title of a work, very neatly gotten up by the publishers, which all lovers of Biography, and especially of the biography of medical men, will be likely to purchase and peruse with pleasure.

Dr. J. C. Warren, late of Boston, attained a very high rank as a surgeon and a teacher; and all the more important facts and incidents of his life will be sought after and preserved, not only by the present generation to whom his name is familiar, but by succeeding generations, so long as the profession itself is cultivated and cherished among men. Dr. Edward Warren, the compiler of this work, had in his possession the most ample materials, and he has used them in such a way as to make two volumes of about 400 pages each, which should be read by every physician in our country.

